# N-substituted benzamides inhibit NF**κ**B activation and induce apoptosis by separate mechanisms

**DOI:** 10.1038/sj.bjc.6690796

**Published:** 1999-11

**Authors:** D Liberg, B Lazarevic, R W Pero, T Leanderson

**Affiliations:** Immunology andMolecular Ecogenetic Groups, Department of Cell and Molecular Biology, Lund University, Sölvegatan 21, Lund, S-223 62, Sweden

**Keywords:** benzamides, apoptosis, NFκB

## Abstract

Benzamides have been in clinical use for many years in treatment against various disorders. A recent application is that as a sensitizer for radio- or chemotherapies. We have here analysed the mechanism of action of N-substituted benzamides using an in vitro system. We found that while procainamide was biologically inert in our system, the addition of a chloride in the 3′ position of the benzamide ring created a compound (declopramide) that induced rapid apoptosis. Furthermore, declopramide also inhibited NFκB activation by inhibition of IκBβ breakdown. An acetylated variant of declopramide, N-acetyl declopramide, showed no effect with regard to rapid apoptosis induction but was a potent inhibitor of NFκB activation. In fact, the addition of an acetyl group to procainamide in the 4′ position was sufficient to convert this biologically inactive substance to a potent inhibitor of NFκB activation. These findings suggest two potential mechanisms, induction of early apoptosis and inhibition of NFκB mediated salvage from apoptosis, for the biological effect of N-substituted benzamides as radio- and chemo-sensitizers. In addition it suggests that N-substituted benzamides are potential candidates for the development of anti-inflammatory compounds using NFκB as a drug target. © 1999 Cancer Research Campaign


British Journal of Cancer (1999) 81(6), 981–988
© 1999 Cancer Research Campaign
Article no. bjoc.1999.0796

### N-substituted benzamides inhibit NFκB activation and induce apoptosis by separate mechanisms

**D Liberg[1], B Lazarevic[1], RW Pero[2] and T Leanderson[1]**

[1]Immunology and [2]Molecular Ecogenetic Groups, Department of Cell and Molecular Biology, Lund University, Sölvegatan 21, S-223 62, Lund, Sweden

**Summary** Benzamides have been in clinical use for many years in treatment against various disorders. A recent application is that as a sensitizer for radio- or chemotherapies. We have here analysed the mechanism of action of N-substituted benzamides using an in vitro system. We found that while procainamide was biologically inert in our system, the addition of a chloride in the 3′ position of the benzamide ring created a compound (declopramide) that induced rapid apoptosis. Furthermore, declopramide also inhibited NFκB activation by inhibition of IκBβ breakdown. An acetylated variant of declopramide, N-acetyl declopramide, showed no effect with regard to rapid apoptosis induction but was a potent inhibitor of NFκB activation. In fact, the addition of an acetyl group to procainamide in the 4′ position was sufficient to convert this biologically inactive substance to a potent inhibitor of NFκB activation. These findings suggest two potential mechanisms, induction of early apoptosis and inhibition of NFκB mediated salvage from apoptosis, for the biological effect of N-substituted benzamides as radio- and chemo-sensitizers. In addition it suggests that N-substituted benzamides are potential candidates for the development of anti-inflammatory compounds using NFκB as a drug target. © 1999 Cancer Research Campaign

**Keywords**: benzamides; apoptosis; NF kB

Apoptosis, or programmed cell death, is a mechanism for physiological elimination of cells during development or tissue reactions. Apoptosis may be triggered via many different environmental stimuli whose intracellular signalling pathways converge at the level of activation of caspases (Cohen, 1997). These cysteine proteases cleave various substrates that are critical for cell integrity, and also release specific nucleases that are responsible for the characteristic DNA fragmentation connected to apoptotic cell death (Enari et al, 1998; Sakahira et al, 1998). The possibility to selectively trigger apoptosis in transformed cells as a strategy for cancer treatment is an attractive approach and the focus of intense drug development efforts.

The NFκB signalling pathway (reviewed in Ghosh et al, 1998) has also been the subject of intense study as a drug target. From its initial description as a transcription factor for the immunoglobulin κ locus (Sen and Baltimore, 1986), this multimember family of transcriptional activators has been implicated in a large number of biological mechanisms in both health and disease (reviewed in Baeuerle and Baltimore, 1996; Baldwin, 1996). Recently, it was also shown that NFκB was involved in the regulation of apoptotic cell death (Beg and Baltimore, 1996; Liu et al, 1996; Van Antwerp et al, 1996; Wang et al, 1996; Wu et al, 1996). Hence, if NFκB activation was blocked, cells that had received an apoptotic stimulus showed a more pronounced response than a corresponding cell population where the NFκB activation pathway was intact (Beg and Baltimore, 1996; Van Antwerp et al, 1996; Wang et al, 1996). This salvage pathway from apoptotic cell death can thus be seen as a potential mechanism for the survival of tumour cells and inhibition of NFκB activation may thus be a means to treat malignant disease.

Benzamides have been exploited in the clinic as anti-emetics, anti-psychotics, anti-arrhythmics, local anaesthetics, anti-inflammatory agents, anti-tumour agents and radio- and chemosensitizers (Stanley and Rotrosen, 1982; Robert-Piessard et al, 1990; Rang and Urban, 1995; Pero et al, 1998). Variants of the drugs have been shown to have several targets like DNA repair, blood micro-circulation, dopamine and hydroxy-tryptamine receptors, and also to induce DNA damage and apoptosis (Stanley and Rotrosen, 1982; Robert-Piessard et al, 1990; Rang and Urban, 1995; Pero et al, 1998). Here, we have investigated the radio/chemo-sensitizing properties of N-substituted benzamide analogues of procainamide. We show that the addition of a chloride to the procainamide aromatic ring structure converted this compound to an apoptosis-inducing agent that also inhibited NFκB activation. The NFκB inhibition could be further potentiated by a N-acetyl substitution that simultaneously blocked the apoptotic effect, indicating that these functions were independent of each other. In accordance with this, the addition of an aromatic N-acetyl group to the procainamide aromatic ring sufficed to create an inhibitor of NFκB activation also in the absence of a chloride substitution. These findings point to two possible molecular mechanisms for the radio/chemo-sensitizing effects of N-substituted benzamides and they are also pertinent for the development of NFκB inhibitory drugs.

### MATERIALS AND METHODS

### Drugs

Procainamide, N-acetyl procainamide, metoclopramide, N-acetyl metoclopramide, declopramide and N-acetyl declopramide were supplied as purified and structurally elucidated hydrochloride salts

*Received 8 March 1999*
*Revised 25 May 1999*
*Accepted 26 May 1999*

*Correspondence to:* T Leanderson by Oxigene Europe AB (Lund, Sweden). The drugs were dissolved in physiological saline for addition to cell cultures.

### Cell culture

Cells (70Z/3) were grown in Iscoves modified Dulbecco's medium supplemented with 7.5% fetal calf serum (FCS), 50 μM 2-mercaptoethanol, fungizone and gentamycine (Gibco). Lipopolysaccharide (LPS) (Difco, *Escherichia coli* 055:B5) was added at 25 μg $ml^{-1}$ where indicated.

### Cell staining and flow cytometry

Cells (70Z/3) were harvested and washed once in phosphate-buffered saline (PBS). All stainings were performed on ice in Hank's balanced salt solution with 10 mM HEPES buffer, 3% FCS and 0.1% azide. The following commercially supplied reagents were used for staining: 7AAD (Sigma), Annexin V-FITC (fluorescein isothiocyanate; Trevigen) and streptavidin–PE (Southern Biotechnology). As an anti-Igκ reagent a monoclonal rat anti-mouse κ (187.1) that had been biotinylated was used and prior to staining with this reagent the cells were treated with a monoclonal anti-Fc receptor antibody (24G2) for 15 min. All analyses were performed in a Becton Dickinson FACSorter using the Cell Quest software package. One representative experiment out of at least three performed is shown where not otherwise stated.

### Nuclear extracts and electrophoretic mobility shift assay

Nuclear extracts from 2.5 × $10^6$ cells were made according to Schreiber et al (1989). Electrophoretic mobility shift assay (EMSA) was performed as previously described (Liberg et al, 1998). In brief, protein was mixed with 1 μg poly-dI-dC (Pharmacia) and 20 000 cpm of $^{32}P$ end-labelled probe in Bind buffer (20 mM sodium phosphate buffer pH 6.0, 10 mM magnesium chloride ($MgCl_2$), 0.1 mM EDTA, 2 mM dithiothreitol, 0.01% NP-40, 0.1 mM sodium chloride (NaCl), 100 μg $ml^{-1}$ bovine serum albumin and 4% Ficoll). The samples were incubated for 30 min at room temperature and separated on a 5% polyacrylamide gel.

### Western blotting

Cytoplasmic extracts were made from 1 × $10^6$ cells by adding 100 μl of lysis buffer (75 mM Tris pH 8, 100 mM NaCl, 5 mM potassium chloride (KCl), 3 mM $MgCl_2$, 2% NP-40 and 0.1 mM phenylmethyl sulphonyl fluoride (PMSF)) and incubating on ice for 5–10 min. The nuclei were pelleted by centrifugation and the supernatants frozen in aliquots at –80°C. A total of 5–10 μl of the extracts were fractionated by sodium dodecyl sulphate polyacrylamide gel electrophoresis (SDS-PAGE) and transferred to a nylon membrane (Hybond-C extra; Amersham). Polyclonal antibodies specific for IκBβ were obtained from Santa Cruz (sc-945). Secondary horseradish peroxidase (HRP)-conjugated antibodies and chemiluminescence ECL reagents were from Amersham. The membranes were blocked for 20 min in PBS-T (PBS and 0.05% Tween-20) with 5% skimmed milk, after which primary antibodies were added in the same buffer. The membranes were washed for 3 × 5 min in PBS-T, after which the secondary antibodies were added again in PBS-T with milk. The membranes were washed as above, and the chemiluminescence reaction was performed according to the manufacturer's protocol.

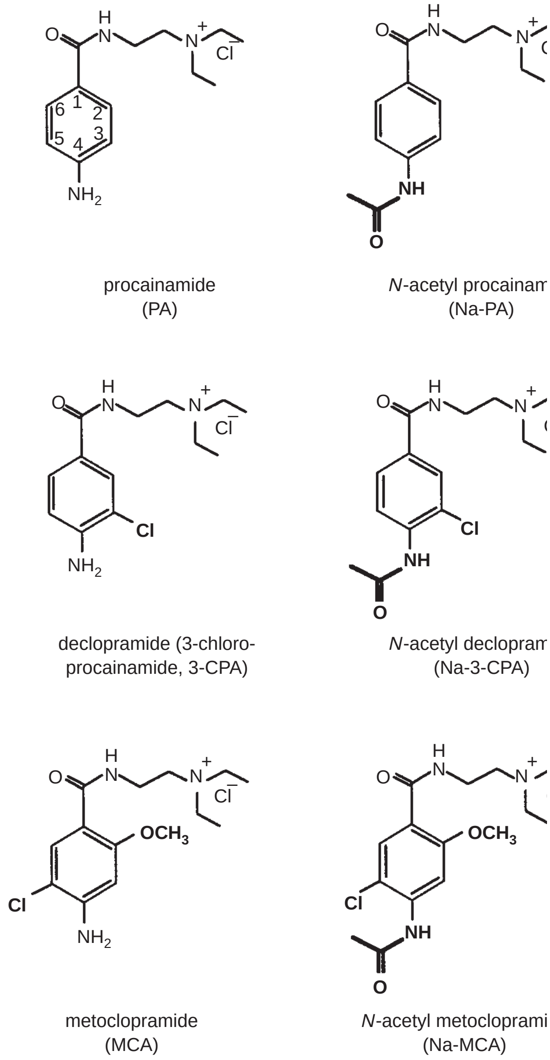

**Figure 1** Structures of procainamide and the benzamide derivatives used in this study. Deviations from the procainamide structure are indicated with bold lines and letters. The abbreviations used throughout this paper are indicated in parenthesis

### RESULTS

### Rapid induction of apoptosis by N-substituted benzamides

To analyse the effects of N-substituted benzamides we synthesized a set of benzamide hydrochloride salt derivatives using procainamide (PA) as a basic structure. These analogues are shown in Figure 1 together with the abbreviations used throughout this paper. In all analogues the side-chain was kept unaltered while various active groups were added to the ring structure. Some of these molecules, e.g. declopramide (3-CPA) and metoclopramide (MCA) have previously been studied in vitro, in vivo and in clinical models (Kjellen et al, 1989, 1995; Lybak et al, 1990; Lybak and Pero, 1991; Hua et al, 1995, 1997; Werning et al, 1995; Hua and Pero, 1997; Olsson et al, 1997; Pero et al, 1998), and have been shown to be biologically active.

Our in vitro model in this study was the well-characterized murine pre-B-cell line 70Z/3 (Paige et al, 1978; Perry and Kelley, 1979). This cell line contains a silent but functionally rearranged immunoglobulin (Ig) κ locus that will be transcribed upon nuclear

 *© 1999 Cancer Research Campaign*

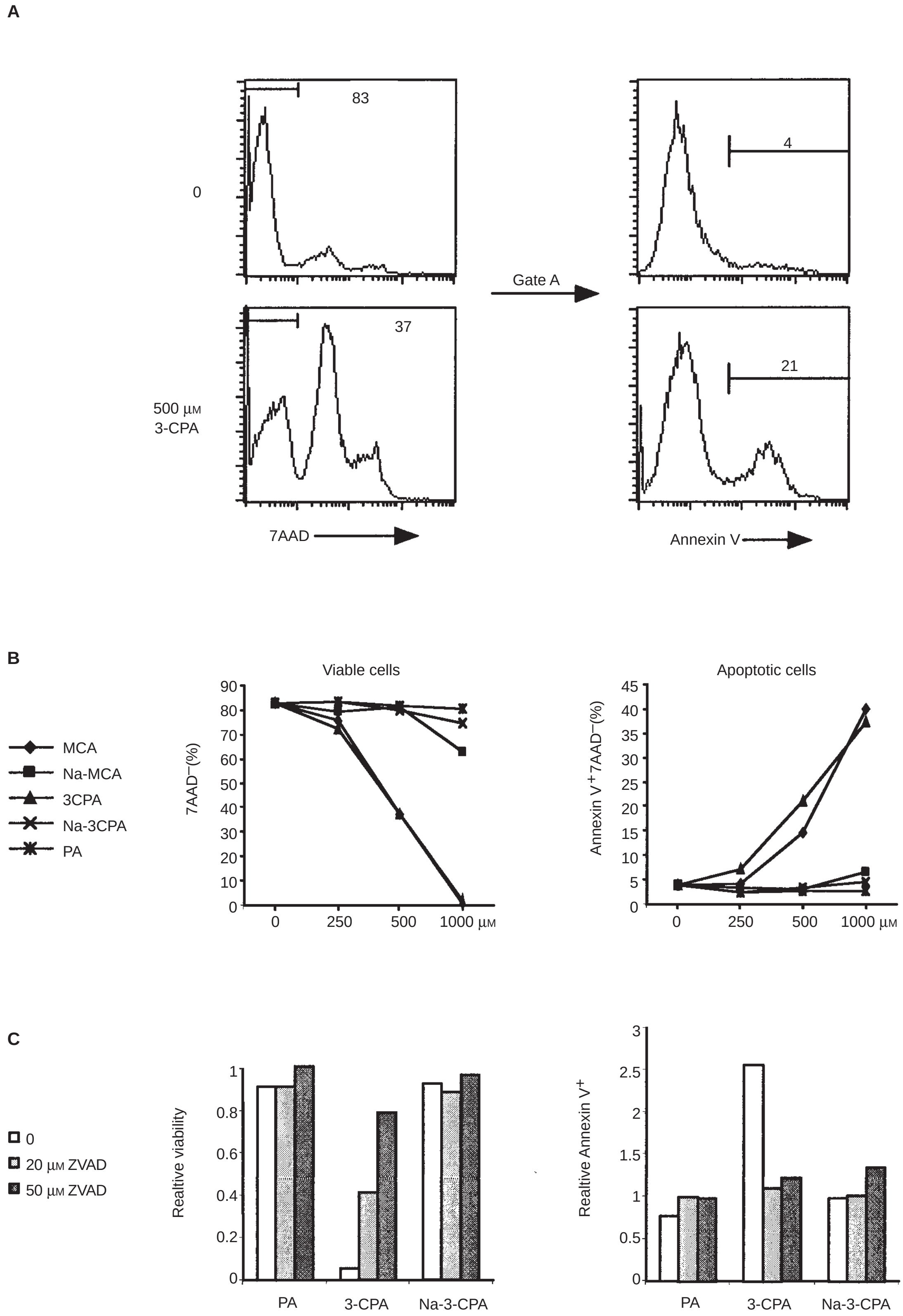

**Figure 2** (**A**) Experimental setup for apoptosis analysis. The figure shows an example of analysis of 70Z/3 cells treated with 500 μM 3-CPA for 20 h. The 7AAD negative (viable) cells were gated (gate A) and analysed for AnnexinV expression. (**B**) Decrease in viable cell number ($7AAD^-$) and increase in apoptosis induction ($7AAD^-$/$AnnexinV^+$) after treatment of 70Z/3 cells with the indicated drugs for 20 h. (**C**) A caspase 1, 3 and 4 inhibitor (ZVADfmk) counteracts apoptosis induction of 3-CPA. Cells were pretreated with ZVADfmk for 1 h before addition of 500 μM of the indicated drugs and further incubation for 17 h. The graphs show relative viability (%$7AAD^-$ of drug-treated cells/%$7AAD^-$ of untreated cells) and relative AnnexinV staining (%$AnnexinV^+7AAD^-$ of drug-treated cells/% $AnnexinV^+7AAD^-$ of untreated cells) for each concentration of ZVADfmk. One representative experiment out of 2 is shown

*© 1999 Cancer Research Campaign* 

**Table 1**

| Treatment at day 0 | % 7AAD⁻ cells | | |
|---|---|---|---|
| | Day 1 | Day 2 | Day 3 |
| None | 92 | 84 | 73 |
| 500 µM PA | 92 | 87 | 78 |
| 500 µM Na-3-CPA | 92 | 89 | 81 |

Na-3-CPA did not induce delayed apoptosis in 70Z/3 cells. 70Z/3 cells were incubated with the indicated compounds and analysed for the induction of cell death using the 7AAD vital dye at the indicated time points. One representative experiment out of two is shown.

translocation of the NFκB transcription factor (Paige et al, 1978; Rosoff et al, 1984; Rosoff and Cantley, 1985; Sen and Baltimore, 1986 and below). For the detection of viable cells we used the vital dye 7-amino-actinomycin D (7AAD). With this reagent viable cells are $7AAD^-$, while $7AAD^+$ cells tend to be distributed in two separate populations. The brightest staining population corresponds to necrotic/late apoptotic cells while the population with intermediate fluorescence intensity corresponds to apoptotic cells (Schmid et al, 1994; Philpott et al, 1996). To quantitate early apoptosis we also measured the percentage of AnnexinV-positive cells in the $7AAD^-$ population (Koopman et al, 1994). AnnexinV recognizes phosphatidyl serine, which is exposed on the cell surface early during apoptosis due to loss of membrane asymmetry. Thus, using this approach we can obtain two independent, quantitative measurements of apoptosis from the same sample. The assay system is illustrated in Figure 2A for untreated and 3-CPA treated 70Z/3 cells.

We next investigated the effects of the different benzamide derivatives on induction of apoptosis in 70Z/3 cells. The data after 20 h of treatment are shown in Figure 2B. Both the percentages of $7AAD^-$ (viable) cells and of AnnexinV-positive cells in the $7AAD^-$ cell population (early apoptotic cells) are indicated. As predicted, an inverse correlation between these two parameters could be observed. With regard to the benzamides, no induction of apoptosis could be detected by PA. The addition of one chloride atom to the 3′ position of the benzamide ring resulted in a compound (3-CPA) that showed a dose-dependent, pro-apoptotic effect on 70Z/3 cells from 250 to 500 µM concentration. This finding supports previous investigations where the pro-apototic effect of N-substituted benzamides on HL-60 and K562 cells were investigated (Pero et al, 1998). The addition of a methoxy group to the 2′ position of the benzamide ring structure together with the

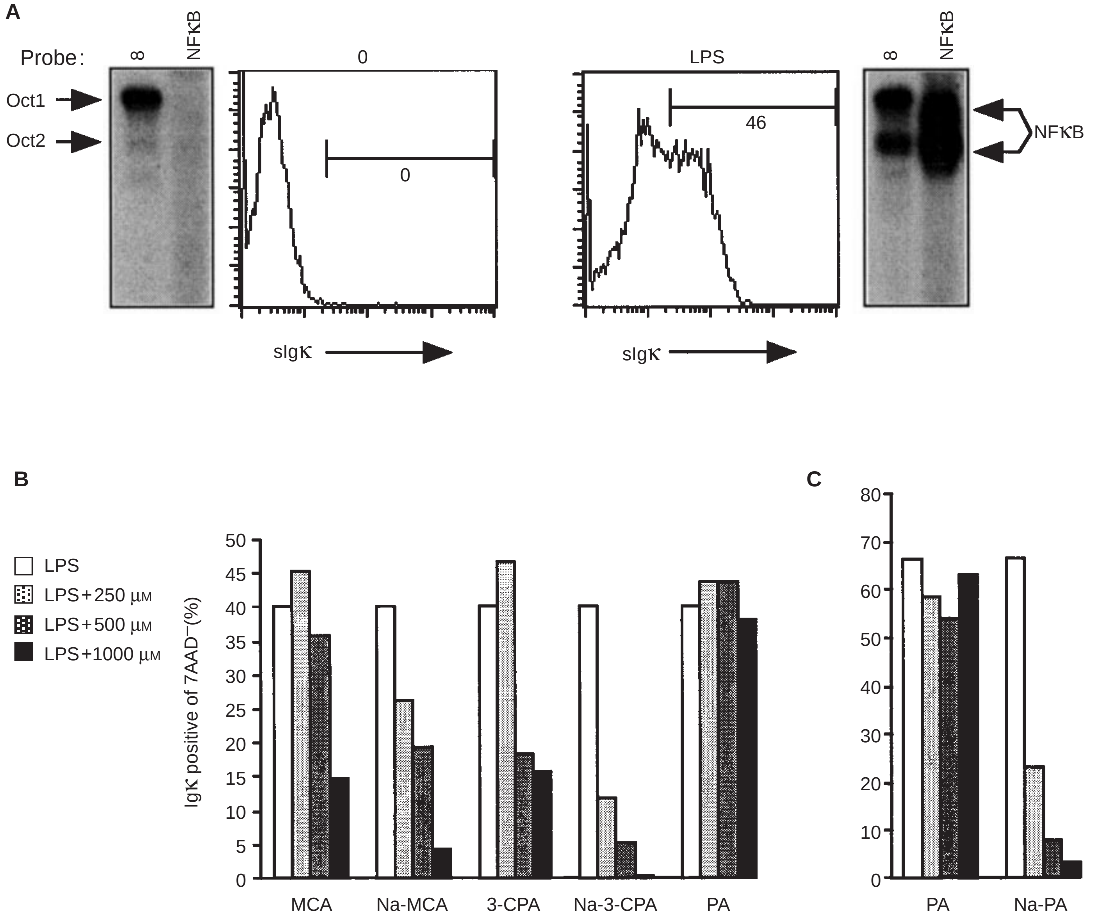

**Figure 3** (**A**) Experimental setup for analysis of Igκ inhibition. 70Z/3 cells were treated with drugs for 5 h before LPS addition and for another 19 h before analysis. 7AAD⁻ cells were gated for as in Figure 2A analysed for expression of surface Ig. Also shown in the Figure is a band-shift analysis using nuclear protein extracts made from unstimulated and LPS-stimulated 70Z/3 cells and an octamer or a NFκB binding site probe. (**B**) Inhibition of surface Ig expression on 70Z/3 cells by different N-substituted benzamides. The drugs were added 5 h before LPS addition and incubation was continued for 19 h before analysis. (**C**) N-acetylation of procainamide mediates inhibition of LPS-induced surface Ig on 70Z/3 cells

 *© 1999 Cancer Research Campaign*

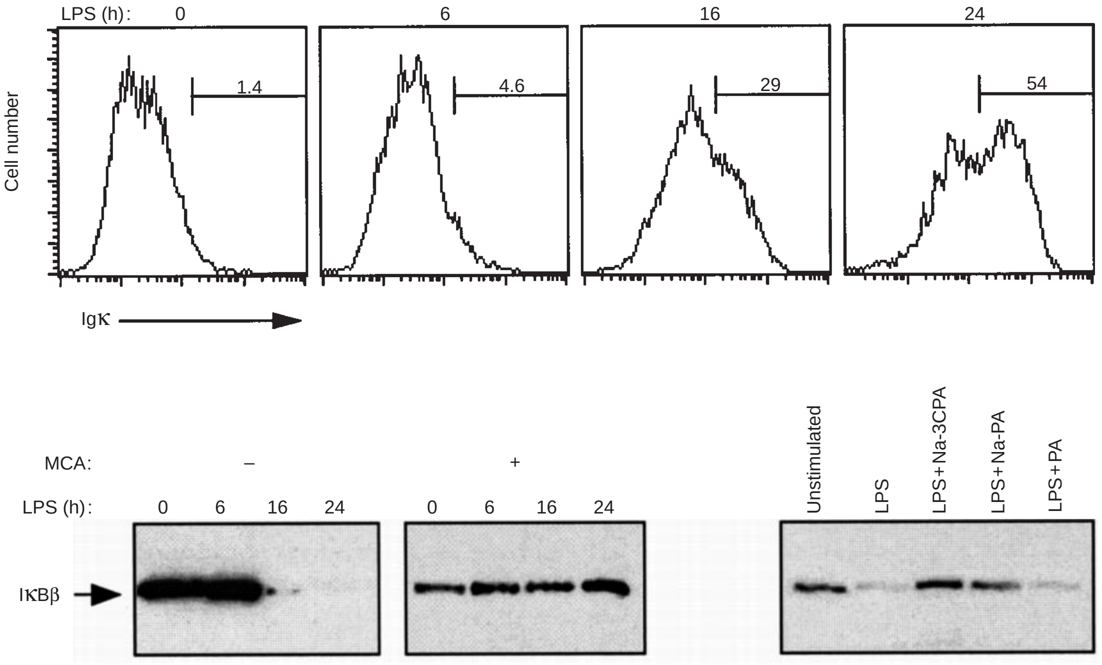

**Figure 4** The kinetics of surface Ig expression on 70Z/3 cells after LPS stimulation correlates with the kinetics of IκBβ degradation. Cytoplasmic extracts were prepared, equalized by Coomassie staining and probed for IκBβ in Western blots. Inclusion of 500 μM MCA, Na-3-CPA or Na-Pa in the cultures inhibited IκBβ breakdown while addition of PA did not chloride and amino substitutions already present in 3-CPA (MCA) did not modify this pro-apoptotic activity. However, the addition of an acetyl group to the 4′ amino group on the benzamide ring (Na-3-CPA; Na-MCA) completely abolished the pro-apoptotic effect of the chloride substitution. Also, PA with the addition of the acetyl group was completely inactive with regard to apoptosis induction (data not shown). To exclude the possibility that Na-3-CPA would induce apoptosis in 70Z/3 cells with different kinetics than 3-CPA, we performed an experiment where we monitored the induction of cell death over a 3-day interval. As shown in Table 1, Na-3-CPA did not increase the number of $7AAD^+$ cells at any of these time points. We conclude from these results that the addition of a chloride atom next to an amino group in the benzamide ring structure correlates with the biological activity as an inducer of rapid apoptosis in 70Z/3 cells. The addition of an acetyl group on the neighbouring aromatic amino group will obliterate this effect.

To further analyse the mechanism of apoptosis induction, we repeated the declopramide experiment in the presence and absence of a caspase 1, 3 and 4 inhibitor (benzyloxycarbonyl-valininalaninyl-aspartyl fluoromethyl ketone (ZVADfmk) (Thornberry et al, 1992; Dolle et al, 1994) (Figure 2C). This caspase inhibitor reduced the induction of apoptosis caused by 3-CPA significantly both when measured as the induction of $7AAD^+$ or $AnnexinV^+$ cells. Thus, the apoptosis-induction activity of 3-CPA involved the activation of at least one of these caspases.

### Inhibition of NFκB activation by N-substituted benzamides

The NFκB signalling pathway has been shown to be able to salvage cells from apoptotic cell death in some experimental systems (Beg and Baltimore, 1996; Liu et al, 1996; Van Antwerp et al, 1996; Wang et al, 1996; Wu et al, 1996). The 70Z/3 cell line has been used extensively to study NFκB activation, and we hence proceeded to investigate the effect of the benzamide derivatives on NFκB activation. As mentioned above, this cell line has a recombined but transcriptionally silent immunoglobulin κ locus, the expression of which can be induced by activators of NFκB such as lipopolysaccharide (LPS) (Paige et al, 1978; Sen and Baltimore, 1986). Ig light-chain expression will lead to assembly of a complete Ig molecule that will be expressed on the cell surface, and therefore, surface staining of 70Z/3 cells for Ig expression presents a convenient measurement for NFκB activation. A typical experiment is shown in Figure 3A. Again, we measure only the surface Ig expression on live cells as defined by lack of 7AAD staining. Analysis of this gate in unstimulated 70Z/3 cells showed that virtually 100% of the cells are negative for surface Ig staining. However, after stimulation overnight with 25 μg $ml^{-1}$ LPS, approximately half of the cells were surface positive for Ig, which correlated with nuclear expression of NFκB as determined by band-shift analysis.

We then proceeded to investigate the effect of the benzamide derivatives on NFκB activation using this assay. The cells were preincubated with different concentrations of the drugs for 5 h, and subsequently LPS was added for an additional 19 h. The cells were stained with 7AAD and anti-Igκ and the number of $7AAD^-κ^+$ cells were quantified. As in the apoptosis assay, PA was completely inactive with regard to inhibition of NFκB induction while the chloride-containing variants (3-CPA and MCA) inhibited approximately 50% of the LPS-induced surface Ig (Figure 3B). More importantly, the acetylated variants (Na-MCA, Na-3-CPA), that were essentially inactive in the apoptosis assay, were even more active on a molar basis, and more or less abolished induction of surface Ig at 1 mM concentration.

Although PA had essentially no NFκB inhibitory activity, the chloride substituted analogues had some activity (3-CPA and MCA) whereas the acetylated plus chloride-substituted

*© 1999 Cancer Research Campaign* 

benzamides (Na-3-CPA and Na-MCA) were more potent with regard to inhibition of NFκB activation. These data suggested that the N-acetyl group in itself could be an active site for NFκB inhibition also in the absence of the chloride substitution. A prediction from this structure/function analysis was that N-acetyl PA, having no chloride substitution (Figure 1), should inhibit surface Ig induction in 70Z/3 cells. Indeed, as shown in Figure 3C, the addition of an aromatic N-acetyl group to PA converted this inactive molecule to a potent inhibitor of NFκB activation. We conclude from these experiments that N-substituted benzamides can be converted to potent inhibitors of NFκB activation if an aromatic N-acetyl group is appropriately introduced.

### N-substituted benzamides inhibit IκBβ degradation in 70Z/3 cells

We next wanted to confirm the effect on NFκB activation by N-substituted benzamides at the biochemical level. The NFκB proteins are sequestered in the cytoplasm by IκB molecules (reviewed in Whiteside and Israel, 1997; Ghosh et al, 1998), in 70Z/3 cells mainly by IκBβ (Whiteside et al, 1997). IκBα and IκBβ interact with the same combinations of Rel-proteins, predominantly p50:p65 and p50:c-Rel complexes (Thompson et al, 1995; Whiteside et al, 1997), but they differ in the kinetics of their breakdown and in the signals to which they respond. While IκBα responds to all known inducers of NFκB, IκBβ is only affected by some, like LPS and IL-1 (Thompson et al, 1995). In addition, while IκBα is phosphorylated and broken down rapidly after stimulation, IκBβ follows a somewhat slower kinetics (Thompson et al, 1995; DiDonato et al, 1996). Furthermore, the IκBα gene is itself a target for NFκB and is thus rapidly resynthesized after NFκB activation (de Martin et al, 1993; Le Bail et al, 1993), while IκBβ levels remain low as long as the signal remains (Thompson et al, 1995). Given this background information, we concentrated on the effects of benzamide treatment on IκBβ. As shown in Figure 4 the kinetics of induction of surface Ig and IκBβ breakdown, as measured by Western blotting, after LPS treatment correlated favourably. If the cells were treated with 500 μm MCA the LPS-induced IκBβ breakdown was inhibited. This finding was also confirmed using Na-3-CPA and Na-PA where both were seen to inhibit IκBβ breakdown. Hence, both the chloride substitution and the N-acetyl group addition to the PA backbone resulted in compounds that inhibited IκBβ breakdown.

### DISCUSSION

In this report we show that N-substituted benzamides can induce rapid apoptosis as well as inhibit NFκB activation in target cells. We also show that altering the substitutions on the benzamide ring structure can skew the biological activity of the benzamides either towards apoptosis-promoting activity or NFκB inhibitory activity. These findings explain the mechanisms involved in the biological activity of N-substituted benzamides as radio/chemo-sensitizers. Furthermore, our data also point to the possibility for a rational design of benzamide analogues that show a high selectivity with regard to biological activities. Since N-substituted benzamides have been in clinical use for many years (Stanley and Rotrosen, 1982), such compounds would be promising candidates to be developed for clinical use.

We have first confirmed previous investigations (Pero et al, 1998) with regard to the apoptosis-induction abilities of the N-substituted benzamides. We were able to show that while PA was completely inactive in this assay, MCA and 3-CPA were both potent inducers of apoptosis in the 500 μM concentration range. The activity was completely inhibited by the addition of a caspase 1, 3 and 4 inhibitor, showing that the mechanism of action involved is an early activation of caspases. The biological activity was dependent on a single aromatic chloride substitution on the benzamide ring which distinguishes 3-CPA from PA, whereas the methoxy group that distinguishes MCA from 3-CPA was inert with regard to apoptosis induction. This is interesting since MCA is already used in the clinic as an antiemetic with both local and systemic effects (Harrington et al, 1983). Furthermore, both MCA and 3-CPA have been implicated as potential sensitizers of radio/chemo-therapies and phase 1–3 clinical trials are in progress (Kjellen et al, 1989, 1995; Lybak et al, 1990; Lybak and Pero, 1991; Hua et al, 1995, Werning et al, 1995; Hua and Pero, 1997). Whether this in vivo activity is due to the ability of direct induction of apoptosis by MCA or 3-CPA, or correlates with their ability to inhibit NFκB activation, remains to be established. Lastly, the addition of the aromatic amino group interferes with the apoptosis-inducing ability of both MCA and 3-CPA. We prefer to interpret this observation by steric hindrance rather than induction of an opposing mechanism, but this assumption remains to be tested experimentally.

Beside their ability to induce apoptosis directly in HL-60 and K562 human leukaemia cell lines (Pero et al, 1998), MCA and 3-CPA have also been shown to inhibit tumour necrosis facter α (TNF-α) production in vivo after LPS administration and the expression of a NFκB reporter gene construct in HeLa cells (Pero et al, 1999). Based on this information we investigated the possibility that these drugs might interfere with the NFκB activation pathway. Indeed, both MCA and 3-CPA inhibited NFκB activation measured as the induction of surface Ig on 70Z/3 cells. However, even at the highest concentrations of these drugs tested the inhibition was not complete and the potent induction of apoptosis made it impossible to further increase the concentrations. Interestingly, the induction of apoptosis in 70Z/3 cells by MCA or 3-CPA did not by itself activate the NFκB salvage pathway (Beg and Baltimore, 1996; Liu et al, 1996; Van Antwerp et al, 1996; Wang et al, 1996; Wu et al, 1996), a feature that might be pertinent for the utilization of these drugs in tumour treatment (data not shown). Adding an N-acetyl group to 3-CPA, creating Na-3-CPA, greatly enhanced the potency of the inhibitory capacity on NFκB activation where complete inactivation of NFκB activity was achieved at 1 mM concentration. The importance of the N-acetyl substitution was confirmed by using Na-MCA in our assay, and even more importantly by the utilization of Na-PA. Hence, the addition of an aromatic chloride to PA creates a compound with caspase activation potential, while the addition of an aromatic N-acetyl substitution converts it to a potent inhibitor of NFκB activation. Because Na-PA and not PA showed high NFκB inhibitory activity, the partial inhibitory activity of NFκB activation seen by MCA and 3-CPA is puzzling. It could be argued that the aromatic chloride substitution adjacent to an aromatic amino substitution could partially replace the conformational specificity of the aromatic N-acetyl substitution at the putative active binding site and hence mediate partial NFκB inhibitory activity. Again, further experimentation is needed to clarify this issue.

With regard to the mechanism of action of the benzamides on NFκB activation we have shown that the compounds act at an early stage by inhibiting IκBβ breakdown. The early activation

 © 1999 Cancer Research Campaign pathway for NFκB has been the object of intense study during recent years. A high molecular weight protein complex has been described that contains two IκB-specific kinases (Chen et al, 1996; Lee et al, 1997), as well as other accessory proteins that are necessary for proper NFκB activation (Chen et al, 1996). The action of the N-substituted benzamides prior to IκB breakdown places their presumptive ligand upstream in the NFκB activation pathway.

The pharmaceutical potential of the compounds described herein is significant. With regard to tumour therapy it can be envisioned that different tumours show different sensitivity for the apoptosis-inducing activity of the benzamides, as well as rely to a various extent on NFκB mediated salvage from apoptosis (Pero et al, 1998, 1999). Hence, the selective use, or combination of, benzamides that have selective properties with regard to these biological pathways may increase the efficacy of treatment of malignant disease together with conventional therapies. Lastly, the inhibition of NFκB activation mediated by the N-substituted benzamides may open a possibility for their development in treatment of inflammatory diseases.

### ACKNOWLEDGEMENTS

This study was supported by grants from the Swedish Cancer Society, the Swedish Medical Research Council, Kock's Foundation, the Crafoord Foundation, the Österlunds Foundation and Oxigene Europe AB.

### REFERENCES

Baeuerle PA and Baltimore D (1996) NF-kappa B: ten years after. *Cell* **87**: 13–20

Baldwin AS Jr (1996) The NF-kappa B and I kappa B proteins: new discoveries and insights. *Annu Rev Immunol* **14**: 649–683

Beg AA and Baltimore D (1996) An essential role for NF-kappaB in preventing TNF-alpha-induced cell death. *Science* **274**: 782–784

Chen ZJ, Parent L and Maniatis T (1996) Site-specific phosphorylation of IκBα by a novel ubiquitination-dependent protein kinase activity. *Cell* **84**: 853–862

Cohen GM (1997) Caspases: the executioners of apoptosis. *Biochem J* **326**: 1–16

de Martin R, Vanhove B, Cheng Q, Hofer E, Csizmadia V, Winkler H and Bach FH (1993) Cytokine-inducible expression in endothelial cells of an I kappa B alpha-like gene is regulated by NFκB. *Embo J* **12**: 2773–2779

DiDonato J, Mercurio F, Rosette C, Wu-Li J, Suyang H, Ghosh S and Karin M (1996) Mapping of the inducible IκB phosphorylation sites that signal its ubiquitination and degradation. *Mol Cell Biol* **16**: 1295–1304

Dolle RE, Hoyer D, Prasad CV, Schmidt SJ, Helaszek CT, Miller RE and Ator MA (1994) P1 aspartate-based peptide alpha-((2,6-dichlorobenzoyl)oxy)methyl ketones as potent time-dependent inhibitors of interleukin-1 beta-converting enzyme. *J Med Chem* **37**: 563–564

Enari M, Sakahira H, Yokoyama H, Okawa K, Iwamatsu A and Nagata S (1998) A caspase-activated DNase that degrades DNA during apoptosis, and its inhibitor ICAD. *Nature* **391**: 43–50

Ghosh S, May MJ and Kopp EB (1998) NFκB and rel proteins: evolutionarily conserved mediators of immune responses. *Annu Rev Immunol* **16**: 225–260

Harrington RA, Hamilton CW, Brogden RN, Linkewich JA, Romankiewicz JA and Heel RC (1983) Metoclopramide. An updated review of its pharmacological properties and clinical use. *Drugs* **25**: 451–494

Hua J and Pero RW (1997) Toxicity, antitumor and chemosensitizing effects of 3-chloroprocainamide. *Acta Oncol* **36**: 811–816

Hua J, Olsson A, Sheng Y and Pero RW (1995) Acidic and neutralized metoclopramide formulations sensitize ionizing radiation induced cytotoxicity in a human lung adenocarcinoma xenografted to scid mice. *Anticancer Drugs* **6**: 451–455

Hua J, Olsson AR and Pero RW (1997) Neutral metoclopramide sensitizes cytotoxicity induced by ionizing radiation in SCID mice xenografted with a human brain astrocytoma. *Int J Cancer* **73**: 871–874

Kjellen E, Wennerberg J and Pero R (1989) Metoclopramide enhances the effect of cisplatin on xenografted squamous cell carcinoma of the head and neck. *Br J Cancer* **59**: 247–250

Kjellen E, Pero RW, Brun E, Ewers SB, Jarlman O, Knoos T, Malmstrom P, Tennvall J, Killander D, Olsson A, et al. (1995) A phase I/II evaluation of metoclopramide as a radiosensitiser in patients with inoperable squamous cell carcinoma of the lung. *Eur J Cancer* **31A**: 2196–2202

Koopman G, Reutelingsperger CP, Kuijten GA, Keehnen RM, Pals ST and van Oers MH (1994) Annexin V for flow cytometric detection of phosphatidylserine expression on B cells undergoing apoptosis. *Blood* **84**: 1415–1420

Le Bail O, Schmidt-Ullrich R and Israel A (1993) Promoter analysis of the gene encoding the IκB-α/MAD3 inhibitor of NFκB: positive regulation by members of the rel/NFκB family. *Embo J* **12**: 5043–5049

Lee FS, Hagler J, Chen ZJ and Maniatis T (1997) Activation of the IκB alpha kinase complex by MEKK1, a kinase of the JNK pathway. *Cell* **88**: 213–222

Liberg D, Sigvardsson M, Bemark M and Leanderson T (1998) Differentiation-specific, octamer-dependent costimulation of kappa transcription. *J Immunol* **160**: 3899–3907

Liu ZG, Hsu H, Goeddel DV and Karin M (1996) Dissection of TNF receptor 1 effector functions: JNK activation is not linked to apoptosis while NFκB activation prevents cell death. *Cell* **87**: 565–576

Lybak S and Pero RW (1991) The benzamide derivative metoclopramide causes DNA damage and inhibition of DNA repair in human peripheral mononuclear leukocytes at clinically relevant doses. *Carcinogenesis* **12**: 1613–1617

Lybak S, Kjellen E, Wennerberg J and Pero R (1990) Metoclopramide enhances the effect of ionizing radiation on xenografted squamous cell carcinoma of the head and neck. *Int J Radiat Oncol Biol Phys* **19**: 1419–1424

Olsson AR, Hua J, Sheng Y and Pero RW (1997) Neutral metoclopramide induces tumor cytotoxicity and sensitizes ionizing radiation of a human lung adenocarcinoma and virus induced sarcoma in mice. *Acta Oncol* **36**: 323–330

Paige CJ, Kincade PW and Ralph P (1978) Murine B cell leukemia line with inducible surface immunoglobulin expression. *J Immunol* **121**: 641–647

Pero RW, Olsson A, Amiri A and Chaplin D (1998) Multiple mechanisms of action of the benzamides and nicotinamides as sensitizers of radiotherapy: opportunities for drug design. *Cancer Detect Prev* **22**: 225–236

Pero RW, Axelsson B, Siemann D, Chaplin D and Dougherty G (1999) Newly discovered antiinflammatory properties of benzamides and nicotinamides. *Mol Cell Biochem* **193**: 119–125

Perry RP and Kelley DE (1979) Immunoglobulin messenger RNAs in murine cell lines that have characteristics of immature B lymphocytes. *Cell* **18**: 1333–1339

Philpott NJ, Turner AJ, Scopes J, Westby M, Marsh JC, Gordon-Smith EC, Dalgleish AG and Gibson FM (1996) The use of 7-amino actinomycin D in identifying apoptosis: simplicity of use and broad spectrum of application compared with other techniques. *Blood* **87**: 2244–2251

Rang HP and Urban L (1995) New molecules in analgesia. *Br J Anaesth* **75**: 145–156

Robert-Piessard S, Lebout G and Courant J (1990) Activity of N-(4-6-dimethyl-2-pyridinyl)benzamides and derivatives. *Eur J Med Chem* **25**: 9–19

Rosoff PM, Stein LF and Cantley LC (1984) Phorbol esters induce differentiation in a pre-B-lymphocyte cell line by enhancing Na+/H+ exchange. *J Biol Chem* **259**: 7056–7060

Rosoff PM and Cantley LC (1985) Lipopolysaccharide and phorbol esters induce differentiation but have opposite effects on phosphatidylinositol turnover and Ca2+ mobilization in 70Z/3 pre-B lymphocytes. *J Biol Chem* **260**: 9209–9215

Sakahira H, Enari M and Nagata S (1998) Cleavage of CAD inhibitor in CAD activation and DNA degradation during apoptosis. *Nature* **391**: 96–99

Schmid I, Uittenbogaart CH, Keld B and Giorgi JV (1994) A rapid method for measuring apoptosis and dual-color immunofluorescence by single laser flow cytometry. *J Immunol Methods* **170**: 145–157

Screiber E, Matthias P, Muller MM and Schaffner W (1989) Rapid detection of octamer binding proteins with 'mini extracts', prepared from a small number of cells. *Nucleic Acids Res* **17**: 6419

Sen R and Baltimore D (1986) Inducibility of kappa immunoglobulin enhancer-binding protein Nf-kappa B by a posttranslational mechanism. *Cell* **47**: 921–928

Stanley M and Rotrosen J (1982) *The Benzamides: Pharmacology, Neurobiology and Clinical Aspects*. Raven Press: New York

Thompson JE, Phillips RJ, Erdjument-Bromage H, Tempst P and Ghosh S (1995) I kappa B-beta regulates the persistent response in a biphasic activation of NFκB. *Cell* **80**: 573–582

Thornberry NA, Bull HG, Calaycay JR, Chapman KT, Howard AD, Kostura MJ, Miller DK, Molineaux SM, Weidner JR, Aunins J, et al. (1992) A novel heterodimeric cysteine protease is required for interleukin-1 beta processing in monocytes. *Nature* **356**: 768–774

Van Antwerp DJ, Martin SJ, Kafri T, Green DR and Verma IM (1996) Suppression of TNF-alpha-induced apoptosis by NF-kappa B. *Science* **274**: 787–789

© 1999 Cancer Research Campaign 

Wang CY, Mayo MW and Baldwin AS Jr (1996) TNF- and cancer therapy-induced apoptosis: potentiation by inhibition of NFκB. *Science* **274**: 784–787

Werning JW, Stepnick DW, Jafri A, Megerian CA, Antunez AR and Zaidi SI (1995) Metoclopramide enhances the effect of photodynamic therapy on xenografted human squamous cell carcinoma of the head and neck. *Arch Otolaryngol Head Neck Surg* **121**: 783–789

Whiteside ST and Israel A (1997) IκB proteins: structure, function and regulation. *Semin Cancer Biol* **8**: 75–82

Whiteside ST, Epinat JC, Rice NR and Israel A (1997) I kappa B epsilon, a novel member of the I kappa B family, controls RelA and cRel NFκB activity. *Embo J* **16**: 1413–1426

Wu M, Lee H, Bellas RE, Schauer SL, Arsura M, Katz D, FitzGerald MJ, Rothstein TL, Sherr DH and Sonenshein GE (1996) Inhibition of NFκB/Rel induces apoptosis of murine B cells. *Embo J* **15**: 4682–4690

 *© 1999 Cancer Research Campaign*
